# Associations between gestational diabetes and cardiovascular disease largely operate independently of postpartum causal pathways: A population‐based cohort study in England

**DOI:** 10.1111/dom.70210

**Published:** 2025-11-05

**Authors:** Nerys M. Astbury, Katherine Ripullone, Rema Ramakrishnan, Mark Woodwood, Jane E. Hirst

**Affiliations:** ^1^ Nuffield Department of Primary Care Health Sciences University of Oxford Oxford UK; ^2^ National Perinatal Epidemiology Unit, Nuffield Department of Population Health University of Oxford Oxford UK; ^3^ The George Institute for Global Health, School of Public Health Imperial College London London UK; ^4^ The George Institute for Global Health University of New South Wales, Sydney Sydney New South Wales Australia; ^5^ Nuffield Department of Women's & Reproductive Health University of Oxford Oxford UK

**Keywords:** cardiovascular disease, gestational diabetes, population‐based studies, risk factors, risk management

## Abstract

**Background:**

Gestational diabetes mellitus (GDM) is associated with increased risk of developing type 2 diabetes and cardiovascular disease (CVD). Here we explore whether the associations are mediated by development of type 2 diabetes and other CVD risk factors.

**Methods:**

The Exploring Long‐term Outcomes following PrEgnancy affected by GDM (ELOPE‐GDM) study is a population‐based matched cohort study, containing 43 572 records of women diagnosed with GDM matched with 174 288 records of non‐GDM women.

We used Cox proportional hazards models to assess the risk of GDM on CVD, ischemic heart disease (IHD) and stroke/TIA and quantified the proportions of these effects mediated by the progression to type 2 diabetes, hypertension or dyslipidaemia using causal mediation analysis.

**Results:**

There were significant associations between GDM and CVD; (adjusted HR 1.58 (95% CI 1.27–1.97)), IHD (1.83 (1.35–2.49)) and stroke/TIA (1.43 (1.06–1.95)). There were strong associations between GDM and developing type 2 diabetes (OR 13.90 (95% CI 13.19–14.51)), hypertension (1.87 (1.781–1.92)), dyslipidaemia (1.80 (1.76–1.84)) or any of these postpartum mediators (1.67 (1.63–1.71)). However, most of the effect of GDM on CVD was not attributed to the overall mediating effects of type 2 diabetes (36% (95% CI 8%–64%)), hypertension (15% (5%–24%)), dyslipidaemia (37% (18%–55%)) or a combination of these conditions (32% (11%–53%)) which developed after pregnancy.

**Conclusion:**

These findings emphasise the need for comprehensive cardio metabolic screening following a pregnancy affected by GDM.

## 1 INTRODUCTION

Gestational diabetes mellitus (GDM), defined as glucose intolerance with first onset during pregnancy, is one of the most common pregnancy complications and is estimated to affect around 16% of pregnancies in the UK and up to 30% worldwide.^1^, ^2^

GDM is associated with an increased risk of several adverse maternal and neonatal risks, including pre‐eclampsia, preterm birth, caesarean section, stillbirth, large for gestational age and neonatal hyperinsulinemia,^2^, ^3^, ^4^ although early identification and management can reduce the risks.^5^, ^6^

While GDM typically resolves after birth, GDM can affect the risk of developing many other longer‐term health conditions, including type 2 diabetes^7^, ^8^, ^9^, ^10^ and cardiovascular disease.^11^ However, the relationship between GDM and CVD is complex, given they share several risk factors. For example, many women who develop GDM will enter pregnancy overweight or live with obesity, which is an established risk factor for CVD. Other established CVD risk factors, such as type 2 diabetes, hypertension, and dyslipidaemia, are more common after pregnancies affected by GDM.^12^ However, it is unclear whether the increased risk of CVD in women with GDM can be accounted for by these risk factors.

Few studies have explored the role of type 2 diabetes and other CVD risk factors (hypertension, hyperlipidaemia) as mediators in the relationship between GDM and CVD.

Those that have are limited to investigating the mediating role of type 2 diabetes and have reached inconsistent conclusions.^13^, ^14^, ^15^, ^16^ Most previous studies have used traditional approaches to estimate the direct effect of GDM on CVD either comparing effect estimates in those who develop type 2 diabetes and those who do not, or after controlling for type 2 diabetes based on simply adjusting for it as a mediator in a standard regression modelling.^17^ However, this approach may produce invalid results, which may be biased due to failure to adjust for unmeasured mediator‐outcome confounding, interaction between exposure and mediator and presence of intermediate confounding.^18^

By estimating direct and indirect effects of mediating pathways, we can gain insights into the underlying causal processes of the disease aetiology, disentangling the total effects into those attributed to the mediator and those independent of the mediator. In this context, unravelling the relationship will help identify where targeted efforts for interventions to prevent CVD mortality and morbidity should be made to have greatest impact.

In this largest nationally representative cohort of people with GDM in England, with follow‐up of over 20 years, we set out to quantify the risk of GDM for developing CVD. We further aimed to quantify the mediating role of type 2 diabetes, hypertension and dyslipidaemia in these associations using formal mediation analysis.

## 2 METHODS

### 2.1 Design and data sources

The Exploring Long‐term Outcomes following PrEgnancy affected by GDM (ELOPE‐GDM) project is a prospective, community‐based, matched retrospective cohort study to explore long‐term effects of GDM. ELOPE used data from an anonymised research database of patients from English general practices containing over 15 million active and historical individual patient records (QResearch version 47). The QResearch database includes demographic information, medical diagnoses, prescriptions, referrals and laboratory results from the primary care record, which are linked with other data sets including the Hospital Episode Statistics (HES) from NHS Digital and death certificates from the Office for National Statistics.

### 2.2 Ethical approval and consent

The QResearch database was approved by the East Midlands Derby Research Ethics Committee (reference 18/EM/0400), which waived the need for written informed consent for the collection of deidentified patient data. In accordance with this ethical approval, the protocol for this study was reviewed and approved by the QResearch Scientific Advisory Committee (study reference Q149).

### 2.3 Study population

Data were obtained for women aged 13–49 years who had at least one singleton birth recorded in HES during the study period (1 January 2000–31 December 2021) and were registered at a general practice (GP) that contributed to the QResearch database during that time and had been registered for at least 1 year prior to the birth. Since eligibility was determined by presence of a delivery episode in the HES data, which captures all registrable births from 24 weeks onwards, pregnancies that ended in miscarriage or termination before 24 weeks were excluded. Women who had been diagnosed with any form of diabetes or CVD prior to the index pregnancy were excluded from the analysis.

### 2.4 Exposure

Maternal exposure was a diagnosis of GDM, defined as the presence of Read code for GDM in the primary care record in the 40 weeks preceding the delivery (Table S1, Supporting Information), or a diagnosis of GDM in the HES data determined by the presence of ICD‐10 code O244 or O249 in the hospital episode record linked with the index delivery. Clinical guidelines in England recommend that women at risk of developing GDM should be offered a 2‐h oral glucose tolerance test (OGTT) at 26–28 weeks gestation, with the results of this test used to diagnose GDM using NICE diagnostic thresholds.^19^

### 2.5 Comparator group

Records of GDM mothers were matched with four unique records of mothers who had no record of GDM matched on maternal age at delivery and delivery date (±3 months); these matched records made up the control group.

### 2.6 Outcomes of interest

1. Cardiovascular disease (CVD) was defined as the first recorded diagnosis of an ischaemic heart disease (IHD) or a stroke or transient ischaemic attack (TIA).
2. IHD was defined as the first recorded diagnosis of IHD determined as the presence of a Read code for IHD in the primary care record, a hospital admission in the HES data with ICD‐10 code for IHD (I10‐25) as the primary or secondary reason for admission, or primary or secondary cause of death recorded as IHD using ICD‐10 codes in the ONS death record.
3. Stroke or TIA was defined as the first recorded diagnosis of a stroke or TIA, which was determined as the presence of a Read code for stroke or TIA in the primary care record, a hospital admission in the HES data with ICD‐10 for stroke/TIA (G45 or I60‐64) as primary or secondary reason for admission, or the primary cause of death recorded as stroke/TIA using ICD‐10 codes above in the ONS death record.

Read codes for all outcomes of interest are available in Table S2.

### 2.7 Covariates

We constructed directed acyclic graphs (DAGs) (Figure S2) to identify potential confounders and clarify the assumed causal relationships between exposure and outcome, ensuring appropriate covariate adjustment in our analyses.

Data from the primary care records that were extracted and used as covariates in the analysis comprised self‐reported ethnicity (classified as White, Indian, Bangladeshi, Other Asian, Caribbean, Black African, Chinese or Other); area‐based socioeconomic deprivation status using Townsend scores (also known as Townsend Deprivation Index); a UK area‐level measure of material deprivation.^20^ Townsend scores were split into five groups (defined by quintiles) with the first fifth (highest Townsend scores) being most deprived areas and the lowest fifth being the least deprived areas; number of previous pregnancies (grouped into 0, 1, 2, 3, 4 and 5 or more); most recently recorded smoking status (divided into never smoking, ex‐smoker or light [1–9 cigarettes per day], moderate [10–19 cigarettes per day] or heavy smoker [≥20 cigarettes per day]); and pre‐pregnancy BMI (calculated as the mean of any BMI measure in the primary care record in the 2 years preceding the index pregnancy) categorised according to WHO groups.^21^

### 2.8 Statistical analysis

Demographic characteristics of the eligible population by exposure status were reported either as mean (SD) or count and proportions.

We used multivariable Cox proportional hazard models with a robust sandwich estimator with clustering for matched GDM‐control groups and follow‐up timescale in days to estimate hazard ratios (HRs) with 95% CIs comparing GDM with control. We ran a series of nested models: unadjusted, adjustment for demographics (ethnicity, socioeconomic status (using Townsend fifths) and number of previous pregnancies), additional adjustment for smoking status and further adjustment for pre‐pregnancy BMI (“fully adjusted”). We assessed the proportional hazards assumption using Schoenfeld residuals, and the assumption was not violated.

In our primary analyses missing values for Townsend groups were imputed using the median of other patients registered at the same GP practice. Missing pre‐pregnancy BMI and self‐reported ethnicity were imputed by generating 10 datasets using chained equations with effects combined using Rubin's rules.^22^ The sensitivity of the results to different approaches to dealing with missing data was assessed by comparing results from our primary analysis with those obtained using alternative approaches to dealing with the missing data (complete case and missing group indicator). We also conducted sensitivity analysis excluding the records of participants who had less than 2 years of follow‐up.

We used the med4way command in Stata version 17 to decompose the total effect of GDM on the outcomes of interest in the presence of potential mediators (type 2 diabetes, hypertension or dyslipidaemia which developed postpartum).^23^, ^24^ Only records of these mediators recorded for the first time after the index pregnancy and before the diagnosis of the outcomes of interest were used to avoid surveillance bias.^25^ The primary analysis used a set of single mediator models, but in a post hoc analysis, we explored the mediating effect of combining these mediators in a single model. The causal mediation framework decomposes the (overall) total effect of GDM on the outcomes of interest into a Controlled Direct Effect (CDE) and Pure Indirect Effect (PIE). The CDE is an estimate of the effect of exposure on outcome not explained by mediation or interaction with the measured mediators, while PIE estimates the effect due to mediation only. The key assumptions for this counterfactual framework analysis are correct model specification and a stable unit treatment value. Additionally, it is implicitly assumed there is no unmeasured confounding, although this is virtually impossible to rule out in observational analyses.^24^ All analyses were conducted in STATA V 17^26^ with *p* values ≤0.05 considered statistically significant.

## 3 RESULTS

**FIGURE 1:**
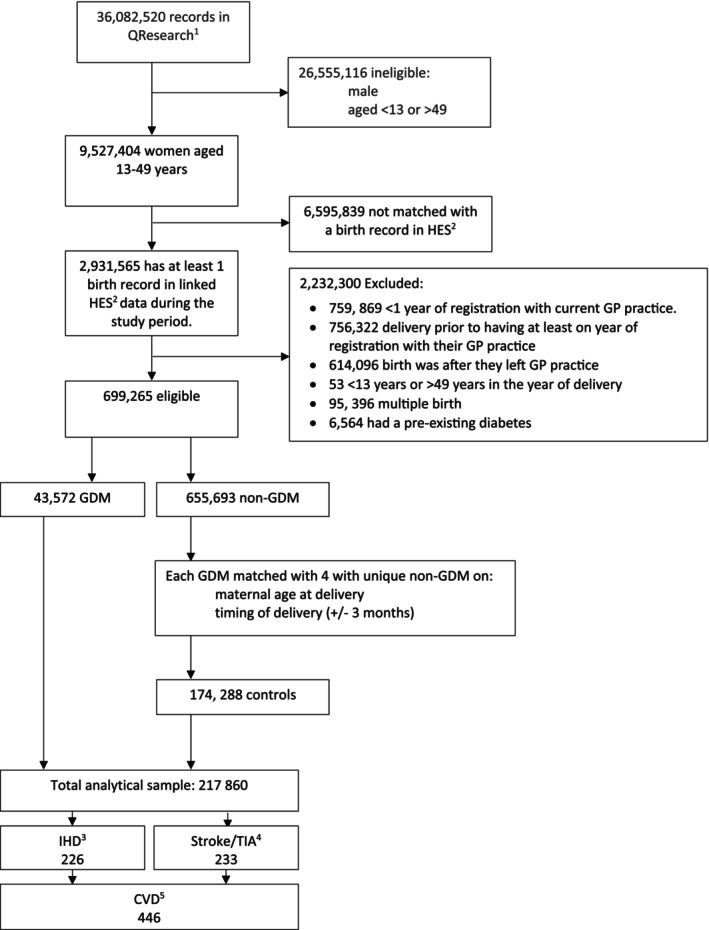
Flowchart of eligible study population. Superscript 1: QResearch is a database of electronic health records from the primary care which are linked with other data sets including the Hospital Episode Statistics (HES) from NHS Digital and death certificates from the Office for National Statistics. Superscript 2: Hospital Episode Statistics (HES) are a dataset containing details of all admissions to NHS hospitals in England, administered by NHS digital. Superscript 3: Ischaemic Heart Disease (IHD). Superscript 4: Transient Ischaemic Attack (TIA). Superscript 5: Cardiovascular disease (CVD).

We identified records of 43 572 eligible women who delivered at least one singleton between 2000 and 2022 and were diagnosed with GDM. These women were matched with 174 288 unique women who had no current or previous history of GDM, who made up the control group resulting in a total analytical sample of 217 860 (Figure 1).

Women who received a diagnosis of GDM had higher pre‐pregnancy weight/BMI, had more previous pregnancies and a greater proportion of women with GDM were from more deprived areas and black and minority ethnic backgrounds than women who did not have a diagnosis of GDM (Table 1).

**TABLE 1:** Demographic characteristics of the analytical sample.

	All, *N* = 217 860	GDM, *N* = 43 572	Control, *N* = 174 288
Age	32.3 (5.2)	32.3 (5.2)	32.3 (5.2)
Age groups, *n* (%)			
14–19 years	1542 (1)	297 (1)	1245 (1)
20–29 years	63 286 (29)	12 612 (29)	50 674 (29)
30–39 years	135 127 (62)	27 048 (62)	108 079 (62)
≥40 years	17 905 (8)	3615 (8)	14 290 (8)
Weight (kg)^a^	70.2 (16.7)	75.8 (19.2)	68.8 (15.7)
BMI (kg/m^2^)^a^	26.2 (6.0)	29.0 (6.6)	25.5 (5.5)
BMI categories, *n* (%)			
>18.5	3804 (2)	433 (1)	3371 (2)
18.5 to ≤25	60 533 (28)	7672 (18)	52 861 (30)
25 to ≤30	34 345 (16)	7863 (18)	26 482 (15)
30 to ≤35	16 728 (8)	5555 (13)	11 173 (6)
>35	11 430 (5)	4581 (11)	6849 (4)
Missing	91 020 (42)	17 468 (40)	73 552 (42)
Previous pregnancies^b^	1.37 (1.54)	1.57 (1.65)	1.32 (1.51)
Previous pregnancy^b^ categories			
0	56 129 (26)	10 212 (23)	45 917 (26)
1	54 208 (25)	10 490 (24)	43 718 (25)
2	29 660 (14)	6520 (15)	23 140 (13)
3	14 744 (7)	3691 (8)	11 053 (6)
4	7360 (3)	2052 (5)	5308 (3)
5 or more	7492 (3)	2014 (5)	5478 (3)
Missing	48 267 (22)	8593 (20)	39 674 (23)
Townsend fifth, *n* (%)^c^			
1st fifth (least deprived)	42 825 (20)	6286 (14)	36 539 (21)
2nd fifth	45 069 (21)	7630 (18)	37 439 (21)
3rd fifth	44 806 (21)	9141 (21)	35 665 (20)
4th fifth	43 302 (20)	9811 (23)	33 491 (19)
5th fifth (most deprived)	41 858 (19)	10 704 (25)	31 154 (18)
Self‐reported ethnicity, *n* (%)^d^			
White	129 486 (59)	19 781 (45)	108 705 (63)
Indian	8664 (33)	2852 (7)	5812 (3)
Pakistani	9453 (32)	3570 (8)	5883 (3)
Bangladeshi	7524 (17)	3986 (9)	3538 (2)
Other Asian	5952 (9)	2078 (5)	3874 (2)
Caribbean	2274 (4)	478 (1)	1796 (1)
Black African	8151	2119 (5)	6032 (3)
Chinese	1766 (1)	451 (1)	1315 (1)
Other	9965 (5)	2189 (5)	7776 (4)
Missing	34 625 (16)	6068 (14)	28 557 (16)

^a^ Calculated as mean of all available measures recorded in the primary care record for each participant in the 2 years prior to index pregnancy.

^b^ Number of previous pregnancies that resulted in a registrable birth (live or stillborn) in the HES delivery record.

^c^ Townsend index fifth is a measure of social deprivation for a geographical area.^39^ A greater Townsend index score indicates a greater degree of deprivation.

^d^ Self‐reported ethnicities.

### 3.1 Risk of cardiovascular disease

During a median follow‐up of 2.9 years (Interquartile interval: 1.1 year to 6.1 years), a total of 233 women had diagnosis of stroke or TIA—226 women had IHD and 13 had both stroke/TIA and IHD, which resulted in 446 women who developed CVD (Figure S1).

**FIGURE 2:**
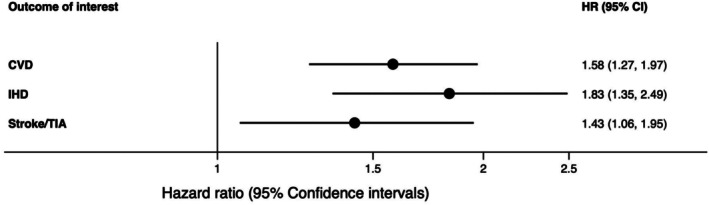
Summary hazard ratios (95% confidence intervals) for the associations between gestational diabetes (GDM) and CVD, IHD and stroke/TIA in fully adjusted models (superscript 1). Superscript 1: Fully adjusted model adjusted for ethnicity, socioeconomic status, smoking status and pre‐pregnancy BMI.

We found significant associations between GDM and each outcome of interest (Figure 2). While including demographics, smoking status and pre‐pregnancy BMI attenuated the effect size, GDM was still associated with substantial and significant increased risk of developing CVD (HR 1.58 (95% CI 1.27–1.97)), IHD (1.83 (1.35–2.49)), and stroke/TIA (1.43 (1.06–1.95)) in the fully adjusted models (Figure 2 and Table S3). The sensitivity analysis excluding those with less than 2 years follow‐up attenuated the estimates, especially for stroke/TIA (Table S4). Further sensitivity analyses using different methods of dealing with missing data did not materially change the overall results (Table S4).

### 3.2 Mediation analysis

We explored the mediating effect of developing type 2 diabetes, hypertension and dyslipidaemia postpartum in separate models, and also the presence of any of these conditions postpartum as potential mediators in the causal pathway between GDM and the outcomes of interest. The first step in this analysis was to run multivariable logistic models to quantify associations between GDM and mediators after controlling for the confounders. This analysis showed that in the fully adjusted models, GDM diagnosis during pregnancy was significantly associated with the risk of developing type 2 diabetes, hypertension and dyslipidaemia after pregnancy. There was also a significant association between GDM and developing any of these conditions postpartum prior to the occurrence of CVD (Table S6).

**FIGURE 3:**
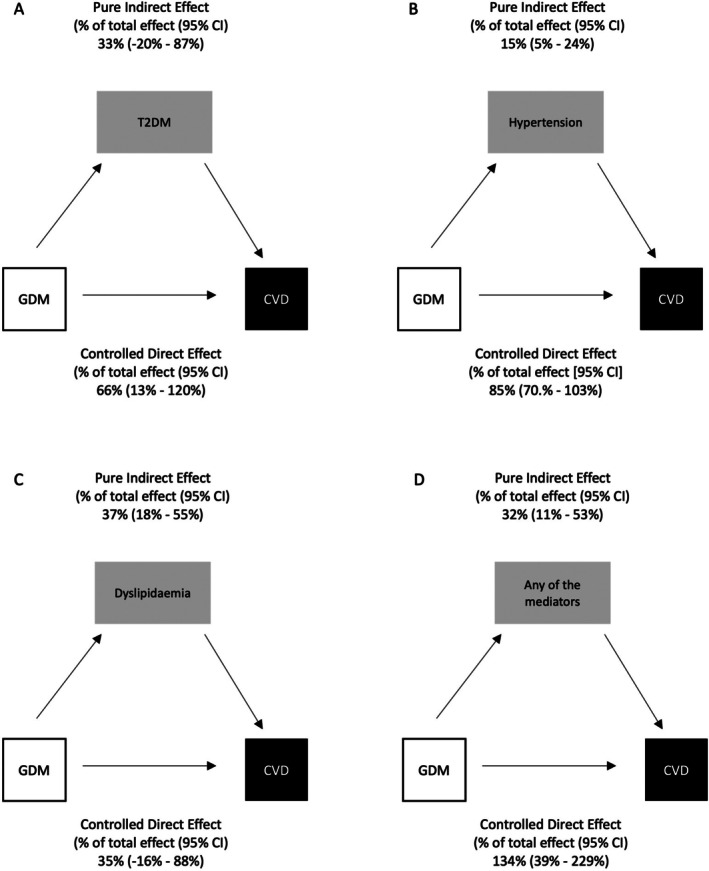
Directed Acyclic graph (DAG) summarising the mediation analysis between gestational diabetes (GDM) and type 2 diabetes (T2DM) (A), hypertension (B), Dyslipidaemia (C) and any of these mediators combined (D) on all‐cause cardiovascular disease (CVD) outcomes.

Four‐way decomposition of the components of the mediation analyses showed the proportion of the total effect mediated by type 2 diabetes was 33% (95% CI −20% to 87%), but this was not statistically significant. In contrast, significant mediation was observed for hypertension (15% (5%–24%)), dyslipidaemia (37% (18%–55%)) and the combined mediators (32% (11%–53%)) (Figure 3). The CDE, the proportion of the total effect attributed to the exposure (GDM) due neither to mediation nor interaction, which can be considered analogous to the proportion of the total effect which would persist if we hypothetically intervened to prevent all postpartum cases of the mediator, suggested that the majority of the effect of GDM on CVD operates through causal pathways that are independent of the subsequent development of type 2 diabetes (66% (95% CI 13–120)), hypertension (85% (95% CI 70%–103%)) and to a lesser extent dyslipidaemia (35% (−16% to 88%)). The CDE for the combined mediators suggested the effect of GDM on CVD operates mostly independently of the effect of the combined postpartum mediator pathways (134% (39%–229%)) (Figure 3).

For the effect of GDM on IHD, there was no significant PIE for type 2 diabetes (43% (−4% to 90%)), but significant PIE was observed for hypertension (50% (14%–86%)), dyslipidaemia (24% (8%–39%)) and any of the mediators (32% (4%–60%)). There were significant CDEs for type 2 diabetes (59% (95% CI 22–95)), but not for hypertension (89% (5% CI 71–107)), dyslipidaemia (18% (−33% to 69%)) or the combined mediators (−25% (−121% to 72%)) (Figure S4).

For the effect of GDM on stroke/TIA, there were no significant PIEs for type 2 diabetes (33% (95% CI −29% to 96%)) or hypertension (38% (−7% to 83%)), despite significant PIE for dyslipidaemia (38% (95% CI 6–71)) and any of the mediators combined (30% (4%–55%)). Nevertheless, there was significant CDE for type 2 diabetes (78% (28%–128%)), dyslipidaemia (83% (15%–151%)), and for any of the mediators combined (80% (26%–134%)), but not for hypertension (47% (−16% to 110%)) (Figure S5).

## 4 DISCUSSION

In this, the largest population‐based cohort of women with GDM in England to date with over 20 years follow‐up, we corroborate previous research reporting GDM is associated with a substantial increased risk of cardiovascular diseases.^11^, ^27^ We expand on previous work by demonstrating that despite elevated risks of developing type 2 diabetes, hypertension and dyslipidaemia in women with GDM, if we intervened with effective interventions to prevent all these mediators from developing, most of the effect of GDM on CVD risk would persist. These findings help inform where targeted interventions for CVD prevention may have greatest impact in women who get GDM.

These findings support our previous findings^27^ and reaffirm a recent systematic review which reported that the risks of cardiovascular and cerebrovascular diseases are higher in women with history of GDM compared with those who do not have GDM.^11^ They also agree with earlier reports that women who develop GDM during pregnancy are at higher risk of CVD in the postpartum period. One study in the UK which reported women with history of GDM had an incidence rate ratio of 1.78 for subsequent development of IHD,^28^ and an analysis from the Nurses' Health Study (USA) reported that women's GDM status (albeit self‐reported) was associated with 59% greater risk of myocardial infarction over a follow‐up period of 25.7 years.^15^ Here we report remarkably similar findings, which were robust to sensitivity analyses which used different approaches to dealing with missing data and excluding those with >2 years follow‐up. Women who had a clinical diagnosis of GDM recorded in their electronic medical record had 60% greater risk of CVD than women who did not have GDM. Together these findings provide evidence supporting the idea that for women who develop GDM who are at increased risk of developing CVD, pregnancy and the postpartum period offer a window of opportunity for cardiovascular prevention. For these women, clinical care should not end at delivery but extend into the postpartum period to incorporate long‐term risk surveillance, lifestyle support and (where appropriate) pharmacological prevention.

While we matched the GDM and control women on age, thus minimising confounding by age, we did see that there were more GDM women from non‐white ethnic backgrounds and more deprived regions. GDM women also had more previous pregnancies and higher pre‐pregnancy bodyweight compared with the control group. These variables have been previously linked with developing GDM.^29^ While we controlled for these factors in our analysis, future work should explore the potential moderating effect of these variables, since previous reports have suggested that pre‐pregnancy obesity could have a conditioning effect, amplifying the elevated risk of CVD seen in women with a history of GDM.^30^

In our present study, we report that GDM was significantly associated with CVD as well as both IHD and stroke or TIA independently; however, differences were evident in the effect estimates for the different sub‐types of CVD, with higher risks seen for IHD than stroke/TIA.

In contrary to our findings, some earlier investigations have reported no significant link between a history of GDM and the occurrence of stroke.^28^, ^31^ These differences may be due to misclassification bias from self‐reported GDM, the small number of events in the population, or too short a follow‐up time to evaluate stroke incidence. These differences in CVD sub‐type (IHD vs. stroke) may be due to the complex pathophysiology which influences some forms of the disease more than others, or to the differences in expected age of onset of the diseases (stroke vs. coronary diseases). Previous studies have suggested associations between GDM and changes in heart structure and function,^32^, ^33^, ^34^ which may go some way to explaining the predominant effect on coronary heart disease seen here and reported in previous studies. But we also found inconsistent mediation patterns across CVD subtypes, with dyslipidaemia mediating 24% of IHD risk and 38% of stroke/TIA risk, despite no significant mediation for type 2 diabetes and hypertension. The discrepancy highlights the potential larger role of lipid metabolism in the development of cerebrovascular disease than cardiovascular disease, which is consistent with previous reports in the literature with meta‐regressions of prevention studies indicating that each 1 mmol/L reduction in LDL cholesterol lowers the overall risk of ischaemic stroke by approximately 20%.^35^ Further research is required to explore how and why changes to lipid subfractions disproportionately contribute to the risk of stroke in women following GDM.

CVD develops over several years or decades, through an accumulation of risk factors.^36^ Although the mechanisms explaining precisely how GDM increases the risk of CVD remain unclear, developing type 2 diabetes, hypertension, dyslipidaemia or other CVD risk factors after pregnancy may act as mediators in the causal relationship between GDM and CVD. Previous studies have reported inconsistent findings regarding the mediating effect of developing type 2 diabetes after pregnancy in the association between GDM and CVD.^13^, ^14^, ^15^, ^16^ We found only one previous large study from Denmark using records from over 1 million women where the authors used structural equation modelling to overcome the limitations in more traditional approaches to explore mediation. Using this approach, they estimated the proportions of the effect of GDM on CVD which could be attributed to developing type 2 diabetes postpartum.^30^ The authors report that less than a quarter of the overall effect of GDM on CVD (23%) could be attributed to the subsequent development of type 2 diabetes.^30^ In the present study we use a similar approach to decompose the components of mediation into its causal pathways, and remarkably, we report comparable results, with 32% of the effect mediated by combined postpartum mediators and a CDE of 134%. A CDE >100% can occur due to interaction (e.g., GDM and mediators may have synergistic effects) or statistical imprecision. Nevertheless, the key interpretation is that the direct effect is substantial, indicating that the effect of GDM on CVD operates largely through causal pathways independent of the combined mediators of interest. This contrasts with what might be expected, given the risk GDM poses for the future development of type 2 diabetes,^7^, ^8^, ^9^, ^10^ as well as the risk that type 2 diabetes has for development of CVD, even before overt disease is evident.^37^ We also expand on previous work by reporting similar effects with respect to the mediating effects of postpartum hypertension and dyslipidaemia, which are associated with GDM and established CVD risk factors.

This is the largest prospective cohort study of women with GDM in England, with over 20 years follow‐up after pregnancy. This large representative sample, with linkages to national hospital episode and death records, has given us the power to undertake the mediation analysis. Other strengths are that all diagnoses of GDM were clinically determined and not self‐reported, which has been a limitation in previous studies. The primary limitation of the mediation analysis is that it is assumed there are no unmeasured confounders, although this is virtually impossible to rule out in observational analyses. Some of these possible unmeasured confounders, for example, diet, physical activity, genetic factors, etc., could explain a proportion of the unexplained direct effect between GDM and CVD. Nevertheless, our findings were robust to adjustment for key measured confounders such as BMI and smoking status. Furthermore, despite the large sample size, the records were limited to patients registered with a GP practice in England which could limit the generalisability of the findings for other countries that use different GDM testing methods, diagnostic thresholds and treatment strategies, or have different population demographics and ethnic mix. Changes in screening, measurement and diagnostic thresholds over the 20‐year study period could have influenced mediator classification, which may result in biases in indirect effects, distortion of direct effects and loss of statistical power. We took exposure, outcome and mediator diagnoses at face value; while this is a good way to discriminate between individuals, it fails to account for differences in the severity of GDM, CVD or type 2 diabetes, hypertension or dyslipidaemia. It is possible that some individuals with sub‐clinical CVD or metabolic dysfunction were misclassified, which could bias mediation estimates. Future research should aim to clarify relationships between more granular metabolic perturbations, including varying patterns in cholesterol subfractions, systolic and diastolic blood pressure measurements and markers of glycaemic control, and their mediating role in the development of cardiovascular disease in women with a history of GDM.

We are aware that there were changes to the clinical guidelines for testing and diagnosis in England which came into effect mid‐way through this study.^38^ However, we used consistent methods to identify GDM throughout based on the standardised Read/ICD‐10 codes, ensuring internal validity despite evolving diagnostic thresholds. While the clinical guidelines after 2015 suggest that GDM be diagnosed using a one‐step two‐hour 75 g oral glucose tolerance test (OGTT), with GDM diagnosed according to NICE diagnostic thresholds,^19^ there are local variations in GDM testing and diagnosis procedures which could introduce further variation to the diagnosis classification.

There are also other limitations with the use of (electronic) health records, as the data used reflect only include data recorded in the medical record in people who seek health care. Data on important variables that are likely to be confounders in the causal pathway between GDM and CVD which are known to contribute to the development of GDM and CVD, such as diet, physical activity and family history, were not available in the medical records, and therefore become “unmeasured confounders” in the analysis, which could produce biased estimates of mediation paths. Other limitations include issues with data quality and completeness. We addressed these by using methods to impute missing values for variables with sizeable amount of missing data (BMI and ethnicity), and the use of sensitivity analysis comparing this approach with other approaches to dealing with missing data and further sensitivity analysis excluding those who had less than 2 years of follow‐up, which also addresses reverse causality due to CVD not being diagnosed at the time of the exposure. While our data provide a relatively long follow‐up for a study of this nature, longer follow‐up (particularly for those who had a more recent delivery) may be required to establish the incidence of diseases like CVD that are more likely to develop with older age. Finally, as with any observational analyses there is a possibility of residual confounding due to unmeasured covariates (e.g., other comorbidities, population density or occupation).

In conclusion, there is substantially elevated risk of CVD after diagnosis of GDM. While a proportion of the association between GDM and CVD can be explained by type 2 diabetes, hypertension and dyslipidaemia that develops postpartum, a greater proportion of this risk is attributed to the direct causal pathway between GDM and CVD. These findings suggest that while interventions in the postpartum period which aim to reduce atherosclerotic risk factors (weight, blood pressure and hypercholesterolemia) may mitigate some of the elevated CVD risk in women with a history of GDM, the greatest benefit may be achieved through clinically effective interventions to prevent GDM implemented either early in pregnancy or even pre‐conception.

## AUTHOR CONTRIBUTIONS

NMA conceived the idea and obtained funding for the project. With the assistance of KR, NMA developed the protocol and statistical analysis plan with input from RR, MW and JEH. Analysis was undertaken by NMA with assistance from KR, RR and MW and all authors provided input on interpretation of results. NMA drafted the manuscript with all authors having input revising the manuscript critically for important intellectual content and read and approved the final submitted manuscript. The corresponding author attests that all listed authors meet authorship criteria and that no others meeting the criteria have been omitted.

## CONFLICT OF INTEREST STATEMENT

MW has been a recent consultant for Amgen and Freeline. All other authors have no conflicts to declare.

## Supporting information


**Figure S1.** Summary of the analytical sample according to development of outcomes of interest.
**Figure S2.** Directed Acyclic graph for the causal pathway.
**Figure S3.** Directed Acyclic graph (DAG) summarising the mediation analysis between gestational diabetes (GDM) and type 2 diabetes (T2DM) (A), hypertension (B), Dyslipidaemia (C) and any of these mediators (D) on ischemic heart disease (IHD) outcomes.
**Figure S4.** Directed Acyclic graph (DAG) summarising the mediation analysis between gestational diabetes (GDM) and type 2 diabetes (T2DM) (A), hypertension (B), Dyslipidaemia (C) and any of these mediators (D) on stroke or transient ischemic attack (TIA) outcomes.
**Table S1.** Primary Care read codes used to identify diagnoses of gestational diabetes mellitus (GDM) in the primary care record.
**Table S2.** Primary Care Read codes used to define the outcomes of interest.
**Table S3.** Associations between gestational diabetes mellitus (GDM) and all‐cause cardiovascular disease (CVD), ischaemic heart disease (IHD) and stroke or transient ischaemic attack (TIA).
**Table S4.** Sensitivity analysis excluding participants with less than 2 years follow‐up from index delivery from the analysis of the associations between gestational diabetes mellitus (GDM) and all‐cause cardiovascular disease (CVD), ischaemic heart disease (IHD) and stroke or transient ischaemic attack (TIA).
**Table S5.** Sensitivity analysis using different approaches to dealing with missing data.
**Table S6.** Association between GDM and type 2 diabetes, hypertension and dyslipidaemia in the postpartum period.

## Data Availability

De‐identified individual participant data that underlie the results reported in this article will be made available, with requests accepted immediately following publication, for proposals that set out to achieve aims specified in a methodologically and scientifically sound protocol that are approved by the QResearch Scientific Advisory Committee (“learned intermediary”), where costs of providing access to the data are covered, where requests are compliant with the legal permissions of QResearch data providers, and QResearch data security requirements are met. Information regarding submission of applications to access data can be found at www.qresearch.org.
