# Supplementary material for: Associations between gestational diabetes and cardiovascular disease largely operate independently of postpartum causal pathways: A population‐based cohort study in England
Source: Diabetes Obes Metab. 2025 Nov 5;28(1):417–26. doi: 10.1111/dom.70210 (PMC12673467; doi:10.1111/dom.70210)
Supplement: Supplementary file 1 — Figure S1. Summary of the analytical sample according to development of outcomes of interest. Figure S2. Directed Acyclic graph for the causal pathway. Figure S3. Directed Acyclic graph (DAG) summarising the mediation analysis between gestational diabetes (GDM) and type 2 diabetes (T2DM) (A), hypertension (B), Dyslipidaemia (C) and any of these mediators (D) on ischemic heart disease (IHD) outcomes. Figure S4. Directed Acyclic graph (DAG) summarising the mediation analysis between gestational diabetes (GDM) and type 2 diabetes (T2DM) (A), hypertension (B), Dyslipidaemia (C) and any of these mediators (D) on stroke or transient ischemic attack (TIA) outcomes. Table S1. Primary Care read codes used to identify diagnoses of gestational diabetes mellitus (GDM) in the primary care record. Table S2. Primary Care Read codes used to define the outcomes of interest. Table S3. Associations between gestational diabetes mellitus (GDM) and all‐cause cardiovascular disease (CVD), ischaemic heart disease (IHD) and stroke or transient ischaemic attack (TIA). Table S4. Sensitivity analysis excluding participants with less than 2 years follow‐up from index delivery from the analysis of the associations between gestational diabetes mellitus (GDM) and all‐cause cardiovascular disease (CVD), ischaemic heart disease (IHD) and stroke or transient ischaemic attack (TIA). Table S5. Sensitivity analysis using different approaches to dealing with missing data. Table S6. Association between GDM and type 2 diabetes, hypertension and dyslipidaemia in the postpartum period. [file DOM-28-417-s001.docx]

**Supplementary Materials**

| **Page No.** |
| --- |
| **Supplementary Figure 1**: Summary of the analytical sample according to development of outcomes of interest 1  **Supplementary Figure 2:** Directed Acyclic Graph for the causal relationships 2  **Supplementary Figure 3:** Directed Acyclic graph (DAG) summarising the mediation analysis between gestational diabetes (GDM) and type 2 diabetes (T2DM) (A), hypertension (B) Dyslipidaemia (C) and any of these mediators (D) on ischemic heart disease (IHD) outcomes. 3  **Supplementary Figure 4:** Directed Acyclic graph (DAG) summarising the mediation analysis between gestational diabetes (GDM) and type 2 diabetes (T2DM) (A), hypertension (B) Dyslipidaemia (C) and any of these mediators (D) on stroke or transient ischemic attack (TIA) outcomes. 4  **Supplementary Table 1:** Primary Care read codes used to identify diagnoses of gestational diabetes mellitus (GDM) in the primary care record 5  **Supplementary Table 2:** Primary Care Read codes used to define the outcomes of interest. 6  **Supplementary Table 3:** Associations between gestational diabetes mellitus (GDM) and all-cause cardiovascular disease (CVD), ischaemic heart disease (IHD) and stroke or transient ischaemic attack (TIA). 8  **Supplementary Table 4: Supplementary Table 4:** Sensitivity analysis excluding participants with less than 2 years follow-up from index delivery from the analysis of the associations between gestational diabetes mellitus (GDM) and all-cause cardiovascular disease (CVD), ischaemic heart disease (IHD) and stroke or transient ischaemic attack (TIA). 9  **Supplementary Table 5:** Sensitivity analysis using different approaches to dealing with missing data 10  **Supplementary Table 6:** Association between GDM and type 2 diabetes, hypertension and dyslipidaemia in the postpartum period. 11 |

**Supplementary Figure 1**: Summary of the analytical sample according to development of outcomes of interest

233

226

13

CVD

446

Stroke/TIA

IHD

**Supplementary Figure 2:** Directed Acyclic Graph

**
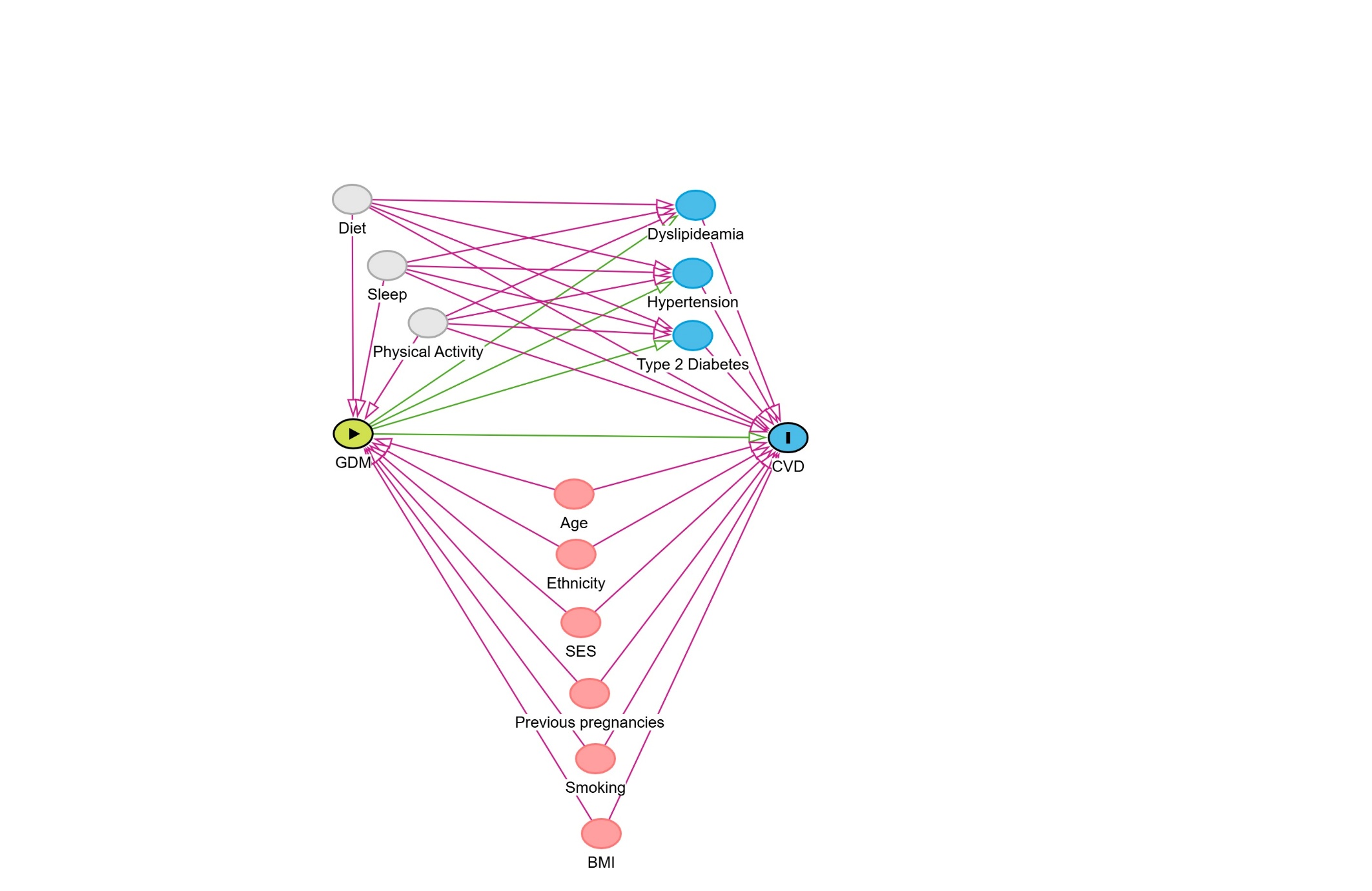
**

**Supplementary Figure 3:** Directed Acyclic graph (DAG) summarising the mediation analysis between gestational diabetes (GDM) and type 2 diabetes (T2DM) (A), hypertension (B), Dyslipidaemia (C) and any of these mediators (D) on ischemic heart disease (IHD) outcomes.

T2DM

IHD

GDM

Controlled Direct Effect

(% of total effect (95% CI)

59% (22% - 95%)

Pure Indirect Effect

(% of total effect (95% CI)

43% (-4% - 90%)

A

Hypertension

IHD

GDM

Controlled Direct Effect

(% of total effect (95% CI)

89% (5% - 107%)

Pure Indirect Effect

(% of total effect (95% CI)

50% (14% - 86%)

B

Any of the mediators

IHD

GDM

Controlled Direct Effect

(% of total effect (95% CI)

-25% (-121% - 72%)

Pure Indirect Effect

(% of total effect (95% CI)

32% (4% - 60%%)

D

Dyslipidaemia

IHD

GDM

Controlled Direct Effect

(% of total effect (95% CI)

18% (-33% - 69%)

Pure Indirect Effect

(% of total effect (95% CI)

24% (8% - 39%)

C

**Supplementary Figure 4:** Directed Acyclic graph (DAG) summarising the mediation analysis between gestational diabetes (GDM) and type 2 diabetes (T2DM) (A), hypertension (B) Dyslipidaemia (C) and any of these mediators (D) on stroke or transient ischemic attack (TIA) outcomes

T2DM

Stroke/TIA

GDM

Controlled Direct Effect

(% of total effect (95% CI)

78% (28% - 128%)

Pure Indirect Effect

(% of total effect (95% CI)

33% (-29% - 96%)

A

Hypertension

Stroke/TIA

GDM

Controlled Direct Effect

(% of total effect [95% CI]

47% (-16.% - 110%)

Pure Indirect Effect

(% of total effect (95% CI)

38% (-7% - 83%)

B

Any of the mediators

Stroke/TIA

GDM

Controlled Direct Effect

(% of total effect (95% CI)

80% (26% - 134%)

Pure Indirect Effect

(% of total effect (95% CI)

30% (4% - 55%)

D

Dyslipidaemia

Stroke/TIA

GDM

Controlled Direct Effect

(% of total effect (95% CI)

83% (15% - 151%)

Pure Indirect Effect

(% of total effect (95% CI)

38% (6% - 71%)

C

**Supplementary Table 1:** Primary Care read codes used to identify diagnoses of gestational diabetes mellitus (GDM) in the primary care record.

| **Gestational diabetes mellitus (GDM)** |
| --- |
| \| **66Ay** \| Gestational diabetes mellitus annual review \| 1 \| - \|  \| \| --- \| --- \| --- \| --- \| --- \| \| **L180** \| Diabetes mellitus during pregnancy/childbirth/puerperium \| 1 \| - \|  \| \| **L1801** \| Diabetes mellitus during pregnancy - baby delivered \| 1 \| - \|  \| \| **L1803** \| Diabetes mellitus during pregnancy - baby not yet delivered \| 1 \| - \|  \| \| **L1808-1** \| Gestational diabetes mellitus \| 1 \| - \|  \| \| **L1809** \| Gestational diabetes mellitus \|  \|  \|  \| |

**Supplementary Table 2:** Primary Care Read codes used to define the outcomes of interest.

| **All-cause cardiovascular disease** | |
| --- | --- |
| **Ischaemic heart disease (IHD)** | **Stroke/TIA** |
| \| I11 \| Hypertensive heart disease \| 1 \| - \|  \| \| --- \| --- \| --- \| --- \| --- \| \| I110 \| Hypertensive heart disease with (congestive) heart failure \| 1 \| - \|  \| \| I119 \| Hypertensive heart disease without (congestive) heart failure \| 1 \| - \|  \| \| I12 \| Hypertensive renal disease \| 1 \| - \|  \| \| I120 \| Hypertensive renal disease with renal failure \| 1 \| - \|  \| \| I129 \| Hypertensive renal disease without renal failure \| 1 \| - \|  \| \| I13 \| Hypertensive heart and renal disease \| 1 \| - \|  \| \| I130 \| Hypertensive heart and renal disease with (congestive) heart failure \| 1 \| - \|  \| \| I131 \| Hypertensive heart and renal disease with renal failure \| 1 \| - \|  \| \| I132 \| Hypertensive heart and renal disease with both (congestive) heart failure and renal failure \| 1 \| - \|  \| \| I139 \| Hypertensive heart and renal disease, unspecified \| 1 \| - \|  \| \| I15 \| Secondary hypertension \| 1 \| - \|  \| \| I20 \| Angina pectoris \| 1 \| - \|  \| \| I200 \| Unstable angina \| 1 \| - \|  \| \| I201 \| Angina pectoris with documented spasm \| 1 \| - \|  \| \| I208 \| Other forms of angina pectoris \| 1 \| - \|  \| \| I209 \| Angina pectoris, unspecified \| 1 \| - \|  \| \| I21 \| Acute myocardial infarction \| 1 \| - \|  \| \| I210 \| Acute transmural myocardial infarction of anterior wall \| 1 \| - \|  \| \| I211 \| Acute transmural myocardial infarction of inferior wall \| 1 \| - \|  \| \| I212 \| Acute transmural myocardial infarction of other sites \| 1 \| - \|  \| \| I213 \| Acute transmural myocardial infarction of unspecified site \| 1 \| - \|  \| \| I214 \| Acute subendocardial myocardial infarction \| 1 \| - \|  \| \| I219 \| Acute myocardial infarction, unspecified \| 1 \| - \|  \| \| I22 \| Subsequent myocardial infarction \| 1 \| - \|  \| \| I220 \| Subsequent myocardial infarction of anterior wall \| 1 \| - \|  \| \| I221 \| Subsequent myocardial infarction of inferior wall \| 1 \| - \|  \| \| I228 \| Subsequent myocardial infarction of other sites \| 1 \| - \|  \| \| I229 \| Subsequent myocardial infarction of unspecified site \| 1 \| - \|  \| \| I23 \| Certain current complications following acute myocardial infarction \| 1 \| - \|  \| \| I230 \| Haemopericardium as current complication following acute myocardial infarction \| 1 \| - \|  \| \| I231 \| Atrial septal defect as current complication following acute myocardial infarction \| 1 \| - \|  \| \| I232 \| Ventricular septal defect as current complication following acute myocardial infarction \| 1 \| - \|  \| \| I233 \| Rupture of cardiac wall without haemopericardium as current complication following acute myocardial infarction \| 1 \| - \|  \| \| I234 \| Rupture of chordae tendineae as current complication following acute myocardial infarction \| 1 \| - \|  \| \| I235 \| Rupture of papillary muscle as current complication following acute myocardial infarction \| 1 \| - \|  \| \| I236 \| Thrombosis of atrium, auricular appendage, and ventricle as current complications following acute myocardial infarction \| 1 \| - \|  \| \| I238 \| Other current complications following acute myocardial infarction \| 1 \| - \|  \| \| I24 \| Other acute ischaemic heart diseases \| 1 \| - \|  \| \| I240 \| Coronary thrombosis not resulting in myocardial infarction \| 1 \| - \|  \| \| I241 \| Dressler syndrome \| 1 \| - \|  \| \| I248 \| Other forms of acute ischaemic heart disease \| 1 \| - \|  \| \| I249 \| Acute ischaemic heart disease, unspecified \| 1 \| - \|  \| \| I25 \| Chronic ischaemic heart disease \| 1 \| - \|  \| \| I250 \| Atherosclerotic cardiovascular disease, so described \| 1 \| - \|  \| \| I251 \| Atherosclerotic heart disease \| 1 \| - \|  \| \| I252 \| Old myocardial infarction \| 1 \| - \|  \| \| I253 \| Aneurysm of heart \| 1 \| - \|  \| \| I254 \| Coronary artery aneurysm and dissection \| 1 \| - \|  \| \| I255 \| Ischaemic cardiomyopathy \| 1 \| - \|  \| \| I256 \| Silent myocardial ischaemia \| 1 \| - \|  \| \| I258 \| Other forms of chronic ischaemic heart disease \| 1 \| - \|  \| \| I259 \| Chronic ischaemic heart disease, unspecified \| 1 \| - \|  \| \| I26 \| Pulmonary embolism \| 1 \| - \|  \| \| I260 \| Pulmonary embolism with mention of acute cor pulmonale \| 1 \| - \|  \| \| I269 \| Pulmonary embolism without mention of acute cor pulmonale \| 1 \| - \|  \| \| I27 \| Other pulmonary heart diseases \| 1 \| - \|  \| \| I270 \| Primary pulmonary hypertension \| 1 \| - \|  \| \| I271 \| Kyphoscoliotic heart disease \| 1 \| - \|  \| \| I272 \| Other secondary pulmonary hypertension \| 1 \| - \|  \| \| I278 \| Other specified pulmonary heart diseases \| 1 \| - \|  \| \| I279 \| Pulmonary heart disease, unspecified \|  \|  \|  \| | \| F4236 \| Amaurosis fugax \| 1 \| - \|  \| \| --- \| --- \| --- \| --- \| --- \| \| Fyu55 \| [X]Other transient cerebral ischaemic attacks+related syndroms \| 1 \| - \|  \| \| G63y0 \| Cerebral infarct due to thrombosis of precerebral arteries \| 1 \| - \|  \| \| G63y1 \| Cerebral infarction due to embolism of precerebral arteries \| 1 \| - \|  \| \| G64 \| Cerebral arterial occlusion \| 1 \| - \|  \| \| G640 \| Cerebral thrombosis \| 1 \| - \|  \| \| G6400 \| Cerebral infarction due to thrombosis of cerebral arteries \| 1 \| - \|  \| \| G641 \| Cerebral embolism \| 1 \| - \|  \| \| G64-1 \| CVA - cerebral artery occlusion \| 1 \| - \|  \| \| G6410 \| Cerebral infarction due to embolism of cerebral arteries \| 1 \| - \|  \| \| G641-1 \| Cerebral embolus \| 1 \| - \|  \| \| G64-2 \| Infarction - cerebral \| 1 \| - \|  \| \| G64-3 \| Stroke due to cerebral arterial occlusion \| 1 \| - \|  \| \| G64z \| Cerebral infarction NOS \| 1 \| - \|  \| \| G64z0 \| Brainstem infarction \| 1 \| - \|  \| \| G64z1 \| Wallenberg syndrome \| 1 \| - \|  \| \| G64z-1 \| Brainstem infarction NOS \| 1 \| - \|  \| \| G64z1-1 \| Lateral medullary syndrome \| 1 \| - \|  \| \| G64z2 \| Left sided cerebral infarction \| 1 \| - \|  \| \| G64z-2 \| Cerebellar infarction \| 1 \| - \|  \| \| G64z3 \| Right sided cerebral infarction \| 1 \| - \|  \| \| G64z4 \| Infarction of basal ganglia \| 1 \| - \|  \| \| G64z-99 \| Cerebral A. occlusion NOS \| 1 \| - \|  \| \| G65 \| Transient cerebral ischaemia \| 1 \| - \|  \| \| G650 \| Basilar artery syndrome \| 1 \| - \|  \| \| G650-1 \| Insufficiency - basilar artery \| 1 \| - \|  \| \| G65-1 \| Drop attack \| 1 \| - \|  \| \| G652 \| Subclavian steal syndrome \| 1 \| - \|  \| \| G65-2 \| Transient ischaemic attack \| 1 \| - \|  \| \| G653 \| Carotid artery syndrome hemispheric \| 1 \| - \|  \| \| G65-3 \| Vertebro-basilar insufficiency \| 1 \| - \|  \| \| G654 \| Multiple and bilateral precerebral artery syndromes \| 1 \| - \|  \| \| G656 \| Vertebrobasilar insufficiency \| 1 \| - \|  \| \| G65-99 \| Transient Ischaemic Attacks \| 1 \| - \|  \| \| G65y \| Other transient cerebral ischaemia \| 1 \| - \|  \| \| G65z \| Transient cerebral ischaemia NOS \| 1 \| - \|  \| \| G65z0 \| Impending cerebral ischaemia \| 1 \| - \|  \| \| G65z1 \| Intermittent cerebral ischaemia \| 1 \| - \|  \| \| G65z-99 \| Transient Ischaemic Attacks \| 1 \| - \|  \| \| G65zz \| Transient cerebral ischaemia NOS \| 1 \| - \|  \| \| G66 \| Stroke and cerebrovascular accident unspecified \| 1 \| - \|  \| \| G66-1 \| CVA unspecified \| 1 \| - \|  \| \| G66-2 \| Stroke unspecified \| 1 \| - \|  \| \| G66-3 \| CVA - Cerebrovascular accident unspecified \| 1 \| - \|  \| \| G667 \| Left sided CVA \| 1 \| - \|  \| \| G668 \| Right sided CVA \| 1 \| - \|  \| \| G66-98 \| Stroke/CVA - undefined \| 1 \| - \|  \| \| G66-99 \| Stroke \| 1 \| - \|  \| \| G6760 \| Cereb infarct due cerebral venous thrombosis, nonpyogenic \| 1 \| - \|  \| \| G6W \| Cereb infarct due unsp occlus/stenos precerebr arteries \| 1 \| - \|  \| \| G6X \| Cerebrl infarctn due/unspcf occlusn or sten/cerebrl artrs \| 1 \| - \|  \| \| Gyu63 \| [X]Cerebral infarction due/unspcf occlusn or sten/cerebrl artrs \| 1 \| - \|  \| \| Gyu64 \| [X]Other cerebral infarction \| 1 \| - \|  \| \| Gyu65 \| [X]Occlusion and stenosis of other precerebral arteries \| 1 \| - \|  \| \| Gyu66 \| [X]Occlusion and stenosis of other cerebral arteries \| 1 \| - \|  \| \| ZV12D \| [V]Personal history of transient ischaemic attack \|  \|  \|  \| |

**Supplementary Table 3:** Associations between gestational diabetes mellitus (GDM) and all-cause cardiovascular disease (CVD), ischaemic heart disease (IHD) and stroke or transient ischaemic attack (TIA).

|  | **All-cause CVD**  HR (95% CI) | **Stroke/TIA**  HR (95% CI) | **IHD**  HR (95% CI) |
| --- | --- | --- | --- |
| **Model 1**  (unadjusted) | 1.89 (1.55, 2.30) | 1.62 (1.22, 2.14) | 2.35 (1.80, 3.09) |
| **Model 2**  (model 1 plus demographics^1^) | 1.79 (1.545, 2.21) | 1.60 (1.19, 2.14) | 2.11 (1.58, 2.80) |
| **Model 3**  (model 2 plus smoking status) | 1.81 (1.47, 2.23) | 1.60 (1.19, 2.15) | 2.15 (1.61, 2.86) |
| **Model 4**  (model 3 plus pre-pregnancy BMI) | 1.58 (1.27, 1.97) | 1.43 (1.05, 1.95) | 1.83 (1.35, 2.49) |

**Supplementary Table 4:** Sensitivity analysis excluding participants with less than 2 years follow-up from index delivery from the analysis of the associations between gestational diabetes mellitus (GDM) and all-cause cardiovascular disease (CVD), ischaemic heart disease (IHD) and stroke or transient ischaemic attack (TIA).

|  | **All-cause CVD**  HR (95% CI) | **Stroke/TIA**  HR (95% CI) | **IHD**  HR (95% CI) |
| --- | --- | --- | --- |
| N  Cases | 133215  409 | 133260  209 | 133319  213 |
| **Model 1**  (unadjusted) | 1.84 (1.50, 2.27) | 1.54 (1.14, 2.07) | 2.35 (1.78, 3.10) |
| **Model 2**  (model 1 plus demographics^1^) | 1.73 (1.39, 2.14) | 1.49 (1.10, 2.05) | 2.08 (1.55, 2.79) |
| **Model 3**  (model 2 plus smoking status) | 1.74 (1.40, 2.17) | 1.50 (1.10, 2.05) | 2.13 (1.59, 2.85) |
| **Model 4**  (model 3 plus pre-pregnancy BMI) | 1.54 (1.22, 1.94) | 1.35 (0.98, 1.88) | 1.82 (1.33, 2.49) |

**Supplementary Table 5:** Sensitivity analysis using different approaches to dealing with missing data.

|  | **Multiple Imputations**  *N* 217630 | **Complete case**  *N* 182022 | **Missing indicator groups**  *N* 217630 |
| --- | --- | --- | --- |
| **All-cause CVD** |  |  |  |
| Cases | 446 | 392 | 446 |
| HR (95% CI) | 1.58 (1.27 – 1.97) | 1.47 (1.17 – 1.85) | 1.59 (1.28 – 1.97) |
| **IHD** |  |  |  |
| Cases | 226 | 201 | 226 |
| HR (95% CI) | 1.83 (1.34 – 2.49) | 1.74 (1.27 – 2.37) | 1.84 (1.37 – 2.47) |
| **Stroke/TIA** |  |  |  |
| Cases | 233 | 203 | 233 |
| HR (95% CI) | 1.443(1.06 – 1.95) | 1.30 (0.93 – 1.80) | 1.44 (1.07 – 1.95) |

**Supplementary table 6**: Association between GDM and type 2 diabetes, hypertension and dyslipidaemia in the postpartum period

|  | **Type 2 diabetes**  OR (95% CI) | **Hypertension**  OR (95% CI) | **Dyslipidaemia**  OR (95% CI) | **Any mediator ^2^**  **OR (95% CI)** |
| --- | --- | --- | --- | --- |
| **Multivariable logistic regression model^1^** | 14.85 (13.64 – 16.16) | 1.75 (1.67 – 1.82) | 1.64 (1.60 – 1.69) | 1.67 (1.63 – 1.71) |

^1^ adjusted for demographics (ethnicity, Townsend fifth and number of previous pregnancies), smoking status and pre-pregnancy BMI

^2^ defined as the presence of type 2 diabetes, hypertension or dyslipidaemia in the postpartum period.
